# Effects of a Long-Term Disturbance on Arthropods and Vegetation in Subalpine Wetlands: Manifestations of Pack Stock Grazing in Early versus Mid-Season

**DOI:** 10.1371/journal.pone.0054109

**Published:** 2013-01-07

**Authors:** Jeffrey G. Holmquist, Jutta Schmidt-Gengenbach, Sylvia A. Haultain

**Affiliations:** 1 White Mountain Research Station, University of California San Diego, Bishop, California, United States of America; 2 Sequoia and Kings Canyon National Parks, Three Rivers, California, United States of America; La Trobe University, Australia

## Abstract

Conclusions regarding disturbance effects in high elevation or high latitude ecosystems based solely on infrequent, long-term sampling may be misleading, because the long winters may erase severe, short-term impacts at the height of the abbreviated growing season. We separated a) long-term effects of pack stock grazing, manifested in early season prior to stock arrival, from b) additional pack stock grazing effects that might become apparent during annual stock grazing, by use of paired grazed and control wet meadows that we sampled at the beginning and end of subalpine growing seasons. Control meadows had been closed to grazing for at least two decades, and meadow pairs were distributed across Sequoia National Park, California, USA. The study was thus effectively a landscape-scale, long-term manipulation of wetland grazing. We sampled arthropods at these remote sites and collected data on associated vegetation structure. Litter cover and depth, percent bare ground, and soil strength had negative responses to grazing. In contrast, fauna showed little response to grazing, and there were overall negative effects for only three arthropod families. Mid-season and long-term results were generally congruent, and the only indications of lower faunal diversity on mid-season grazed wetlands were trends of lower abundance across morphospecies and lower diversity for canopy fauna across assemblage metrics. Treatment x Season interactions almost absent. Thus impacts on vegetation structure only minimally cascaded into the arthropod assemblage and were not greatly intensified during the annual growing season. Differences between years, which were likely a response to divergent snowfall patterns, were more important than differences between early and mid-season. Reliance on either vegetation or faunal metrics exclusively would have yielded different conclusions; using both flora and fauna served to provide a more integrative view of ecosystem response.

## Introduction

Comparisons of persisting versus shorter-term effects of a given long-term disturbance are less common than might be expected; grazing management has provided a good laboratory for such studies, because of detailed, long-term stock use records, the presence of de facto long-term exclosures, and an understanding among managers that long- and short-term grazing effects may differ [1]–[4]. These studies have shown both similarities between long- and short-term effects as well as divergent effects [5], [6]. A significant period of time may be required for indirect effects or other subtle, slow, or complex processes to operate in ecosystems [2], [3], [7] resulting in, for example, short-term increases in nutrients due to grazing eventually transitioning to long-term nutrient decreases [2], [8], which in turn may influence arthropods in complex ways [9], [10]. Some short-term investigations predict long-term species-level effects well but predict assemblage or ecosystem effects poorly (discussion in [2]). In our current work, we contrast effects of pack stock grazing manifested during early and mid-growing season in vulnerable subalpine wetlands. We make use of a landscape-scale, long-term manipulation (see also [11]) of wetland grazing created by the management of recreational pack stock in Sequoia National Park (Sierra Nevada mountains of California, USA) [12]. Effects of mules and other pack stock have been the focus of comparatively few investigations [13]. Our study uses an unusually broad suite of response variables to capture ecosystem response across multiple trophic levels rather than relying on primary producers in isolation [14], [15]. We evaluate responses across the full breadth of Arthropoda in addition to vegetation structural characteristics (see also [16], [17], [18]), allowing detection of both direct and indirect grazing effects. Such indirect effects are under-investigated in assessments of recreational impacts, particularly on larger spatial and temporal scales [19], [20].

Absence of apparent long-term disturbance effects does not render shorter-term effects trivial. Invertebrates may be particularly susceptible to such additional short-term effects, but these impacts may not be easily ascertained, because a) invertebrates have been under-investigated in ecosystem studies in general, and b) short-term effects on invertebrates may not be detected by long-term sampling as a result of masking by dispersal and/or recolonization [21]–[23]. Some studies report mid-season effects on arthropods from grazing or other forms of canopy removal [24]–[26]; canopy removal experiments suggest that univoltine arthropods may be particularly vulnerable to mid-season stock effects [27]. Flowering in the subalpine growing season occurs while stock are present [12], and flower removal can impact butterflies [28] and other nectivores [22], [29], which could result in a feedback loop that reduces populations of both flowering plants and pollinators. Although habitats may recover during the winter, resulting in few apparent long-term effects, it is possible that there are impacts on arthropod assemblages during each growing season that could cascade into vertebrate or upland assemblages [29].

High elevation and high latitude wetlands are valued ecosystem components (e.g., [30]) that have short growing seasons and tend to be vulnerable to anthropogenic disturbance [9], [17], [31], in part because these habitats retain high levels of soil moisture through the growing season and because of slow vegetation growth/regrowth [32]. Conclusions based solely on infrequent, long-term sampling may be particularly likely to be misleading in snow-dominated ecosystems, because the long winter may provide annual recovery periods of nine months or longer that may erase severe, short-term impacts occurring at the height of the abbreviated growing season. Pack stock are an example of a potential disturbance source that coincides with the apex of the growing season in the mountain environment [12], [32], [33]; such timing of disturbance is relatively common in mountain ecosystems [34]. Pack stock graze subalpine wet meadows in short two-three month pulses, followed by a long winter recovery period [12]. Stock are primarily mules and horses, but there are occasional burros or llamas [17], [18], [35]; these animals are used to transport recreationists and materials deep into wilderness areas.

In an initial, one-year study, we sought to determine if these grazing patterns caused lasting effects on terrestrial, epigeal arthropods and associated wetland vegetation, or if the long winters without stock allowed an annual recovery of assemblages from any impacts that occur during summer usage [12]. We sampled in early season only, and stock grazing had moderate long-term effects on vegetation but little apparent effect on arthropod fauna in these wetlands during early season [12]. The primary objective of the current two-year study was to compare a) long-term grazing effects that are manifested in early season before annual usage, with b) the full suite of effects that might become apparent at mid-season, from either the numerous, known components of short-term, annual disturbance (e.g., cropping, hoof punching, feces and urine deposition) or from unknown but possible lasting phenomena that might be detectable later in the stock/growing season but not in early season (e.g., delayed changes in productivity/canopy structure). Our secondary objective was comparison of arthropod assemblage structure, irrespective of grazing, at the beginning and end of the growing season. Higher diversity and abundance might be expected in early season because of high soil moisture or the initial arrival of warmer temperatures, or alternatively in mid-season as a response to better-developed vegetation structure.

## Materials and Methods

### Study Area, Sites, and Design

Wet meadows are saturated with water during much of the year [36], [37], and terrestrial arthropods are abundant and diverse in these habitats [23]. Stock access to subalpine wetlands is generally prohibited by Sequoia National Park until about one month after snowmelt and sometimes substantially longer, depending upon the length of the preceding winter at this elevation (2587–3242 masl). Stock are thus typically present from July-September. We used the same reed grass-dominated (*Calamagrostis muiriana* B.L. Wilson and S. Gray; see [32] for a good image) sites and the same site-scale methodology as in our early season-only study [12]. Grazed wetlands used in the study received a mean of 18.5 (SE  = 4.2) stock nights/ha/year over the last two decades.

Pack stock grazing and associated management practices in Sequoia National Park present an ideal scenario for examination of long-term grazing effects. This work was facilitated by a) the presence of many wet meadows that had been closed to stock for decades that could be paired for contrast with grazed wet meadows with known usage patterns, and b) a controlled opening date for grazing on each wet meadow, so we could sample immediately after greenup, i.e., after there was high quality arthropod habitat, but just before stock grazing. The grazing patterns and management regime enabled us to design what was in essence a subsequent long-term and large-scale experiment (see also [11]). We investigated effects on arthropods and vegetation in subalpine wetlands during early season, and we also sampled after these wetlands had been grazed by stock for the entirety of the growing season.

The study was cast as a 2×2×2 blocked design (Treatment: Control, Grazed; Season: Early, Mid; Year: 2010, 2011) using ten pairs of control and grazed subalpine wet meadows. Each study site had two randomly-selected subsample locations, with two additional randomly-selected subsamples nested within each of the first pair of subsamples. Vegetation in these wetlands typically begins to senesce in mid- to late September [33], and arthropod diversity and abundance decline at this time and remain low through the late, senescent season (Holmquist and Schmidt-Gengenbach, unpublished report). We did mid-season sampling at the end of the growing season, i.e., just before vegetation senescence. Early season comparison of grazed and control sites should reveal long-term effects in isolation from annual, growing season effects. Mid-season comparison of grazed and control sites, in concert with comparison of the same wetlands in early season, should detect any additional impacts manifested during the current growing season. Treatment x Season interactions were thus of particular interest, especially any results that might indicate lack of grazed-control differences in early season but presence of such effects at mid-season. We used a paired design in order to minimize the influence of differences among watersheds, vegetation types, and spatial effects. Site selections were driven by a limited number of sites that had a long history of grazing and that could be paired with control sites, having similar vegetation, that had been closed to grazing for many years. Individual wet meadow pairs were separated by a mean of only 960 m (SE  = 160) and were in the same watersheds, allowing us to sample both meadows of a pair rapidly, before there could be significant meteorological changes. Although individual wet meadow pairs were tightly co-located, meadow blocks were separated by up to 40 km, requiring up to five days of foot travel to sample some groups of sites. The design thus included good replicate dispersion ([38], see also [3]) across a large, wilderness landscape. Distances between grazed and control sites were normally distributed (Lilliefors and Shapiro-Wilks tests, p = 0.24 and 0.94, respectively). Voronoi tessellations and spanning trees were similar for both site categories, and there was no pattern and little roughness apparent for spatial surface models of either control or grazed sites across representative response variables.

We sampled all sites four days or less before grazed sites were opened to pack stock in early season and similarly just before vegetation senescence toward the end of mid-season conditions in both 2010 and 2011. The two years captured varying antecedent conditions, because the winter preceding 2011 sampling produced greater snow water equivalent (SWE  = 131 cm at our Hockett Meadow block; ≈590 cm snow depth) than the winter preceding 2010 sampling (SWE  = 89 cm). Stock opening date at individual meadows is determined by the Park based on soil saturation and vegetation characteristics, so we sampled under similar phenological conditions in each year. Sampling in 2010 began in early July and concluded in early September, whereas 2011 sampling ran from early August through mid-September because of late snowmelt. See [12] for additional details on Park grazing management, vegetation assemblages, site locations, and stock use patterns in individual wet meadows.

### Ethics Statement

A Scientific Research and Collecting permit was obtained from the US National Park Service for work in Sequoia National Park for each year of the study. No protected species were sampled.

### Faunal Methodology

We sampled the wetland canopy assemblage with sweep nets and secondarily targeted ground-dwelling fauna, especially ants, by baiting. Sweep nets are likely the most frequently used device for sampling epigaeic arthropods, can detect sparsely distributed taxa [39], integrate relatively large areas, are easy to transport while backpacking, and do not negatively affect wilderness qualities (see also [12]). Limitations include the potential for efficiency to be influenced by habitat and arthropod assemblage structure, weather, and diel periodicity, vertical distribution, and activity of fauna, [39]. Similarly, baits are the most common tool used to sample ant assemblages [40] and offer many of the same advantages as sweep nets, but there is a higher probability of capture for omnivorous, wide-ranging, or dominant taxa [40], [41]. Differential capture probability can be mitigated by use of baits of differing types [40].

Each sweep sample for a site consisted of 50 standard sweep net sweeps [39], [42] divided between the two subsampling locations at each site and covering a total of 400 m^2^. The collapsible net had a 30.5 cm aperture and mesh size of 0.5×0.75 mm (BioQuip #7112CP). We collected sweep samples prior to the disruption associated with other data collection at the sites, and samples were killed with 99% ethyl acetate [43]. We placed a honey bait at one subsample location and a tuna bait at the other subsample location immediately after sweep netting. The baits were ∼1 cm^2^ portions of honey or tuna that were placed on green construction paper cards and weighted with rocks. After 30 minutes, ants, mites, and other arthropods were removed with forceps and placed in a vial containing 70% ethanol (additional sampling details in [12]).

Sweep samples were sorted in the lab and identified to family (see also [44], [45]) and morphospecies ([46]–[49]; see also [12], [23], [50]). Ants from the bait samples were identified to species. If taxonomic ambiguity (sensu [51]) existed, we used the “distribute parents among children” approach [51] on a per sample basis. Vouchers were stored with the University of California. This study was an investigation targeting specific ecological questions by examining responses across the full breadth of Arthropoda [44], [45]; higher taxonomic resolution was often impractical due to the large collections, an abundance of immature specimens and undescribed species, and because a number of groups await revision.

### Vegetation and Physical Data

We estimated percent bare ground, percent green, standing brown (senescent), and litter cover, as well as canopy height and litter depth on each of the subsample locations. Such coarse vegetation parameters are effective in detecting pack stock impacts on vegetation assemblages [17], [52], [53]. Cover categories were estimated with a point-intercept transect (20 points) centered in each subsample location and randomly oriented. We measured canopy height and litter depth at two random locations within each subsample. Vegetation variables were thus means of two or four measurements at each site.

Air temperature and average wind speed were recorded midway between the two subsample locations with a Kestrel 3000 digital meter in order to verify that similar meteorological conditions obtained between paired grazed and control wetlands. We used a pocket penetrometer (Ben Meadows) to estimate soil strength at each of the locations used for canopy height and litter depth measurement, and the average of these four estimates was the site mean.

### Analysis

We examined the influences of grazing, season, and year on invertebrate assemblages and vegetation structure with both uni- and multivariate approaches. Univariate analyses were 2×2×2 blocked ANCOVAs (df  = 49) using a general linear model in SYSTAT 12. Analysis of site elevation as a covariate was necessary because elevation differed by treatment but could not be affected by the treatment [21], [54] (although mean elevation differences between grazed and control wet meadows were <60 m [12]). Faunal metrics included order and family abundances, family and morphospecies richness and dominance, evenness (probability of interspecific encounter, PIE, [55]), expected number of species scaled to the number of individuals in the sample with the fewest individuals (*E*(*S_18_*); [55], [56]), and percentages of predators, herbivores, and more- and less-motile taxa. Use of *E*(*S*) is valuable, because samples with larger numbers of individuals will tend to have more species, even if all samples represent equal effort and are collected from the same assemblage. We calculated *E*(*S_18_*), and PIE using the application Diversity. Metrics that showed departures from normality (Lilliefors tests; [57]) or heteroscedasticity (*F*_max_ and Cochran's tests; [58]), were modified with square-root transformations ((y)^0.5^+(y+1)^0.5^) of proportional data and log transformations (log (y+1)) of all other data such that parametric assumptions were met. Substitutions were not made for cells with missing values. We used G*Power [59], our known sampling design and sample size, and the standard a priori estimate for effect size of 0.5 [60] to estimate power a priori. Estimated power was high (0.98). We used the sequential Bonferroni adjustment [61] and the application MacBonferroni to calculate alternative, conservative probability values for an overall family-wise error rate of 0.05 across suites of ANCOVAs.

We also used multivariate analyses in order to detect patterns as a function of study factors, across both family and morphospecies matrices, that might not emerge via univariate tests of individual taxa or assemblage metrics (only univariate methods were used in the earlier study [12]). We used hierarchical, polythetic, agglomerative cluster analyses to group samples based upon similarities in response patterns [62], [63]. Differences among groups were then assessed with MRPP using both a priori and a posteriori groupings [62], [63]. We also performed permutational analyses of dispersion, because dispersion patterns of observations have the potential to influence MRPP results. Aphid morphospecies were excluded from multivariate analyses, because of difficulty in distinguishing individual aphid morphospecies across samples. We performed cluster and MRPP analyses using PC-ORD 6 [62], [63] and analyses of dispersion using PERMDISP2 software developed by MJ Anderson (see also [64], [65]). Response and explanatory matrices contained all sites. The response matrices of families and morphospecies included taxa that we sampled at three or more sites (62 families and 120 morphospecies; [62], [63] but see [66]). These matrices were relativized by maximum abundance for each family or morphospecies. The final family primary matrix had a coefficient of variation of 56%, and 71% of the cells contained zeros, and the primary morphospecies matrix had a CV of 45% and 81% zeros. The Sørensen (Bray-Curtis) distance measure [63] was used because this measure is superior for sparse data (with many zeros, as often occurs in taxonomic matrices), retaining sensitivity to heterogeneity without being overly sensitive to outliers [63]. We used group average linkage for the cluster analysis. Treatment and Season coding variables from an explanatory matrix were used for the initial cluster analyses, and an additional group membership variable based on the four highest level groups from the resulting dendrograms, versus initially coded factors, was added to the explanatory matrix for both families and morphospecies. We used MRPP, including pairwise comparisons, with both the initial coding variables and the additional group membership variables. The distance matrices were rank-transformed prior to MRPP. The permutational dispersion analyses were based on 9,999 permutations and used the same datasets and distance measure used for MRPP without additional transformations or other data modifications.

## Results

### Vegetation and Physical

**Figure 1:**
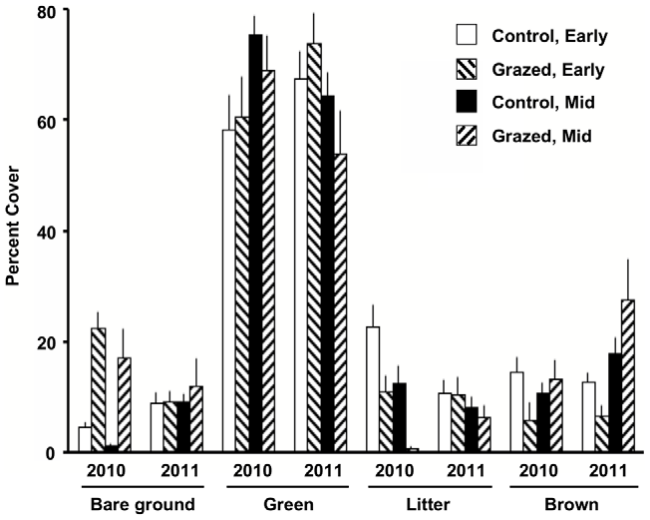
Means (SEs) for percent cover components as a function of grazing treatment and season. See Table 1 for test results.

**Figure 2:**
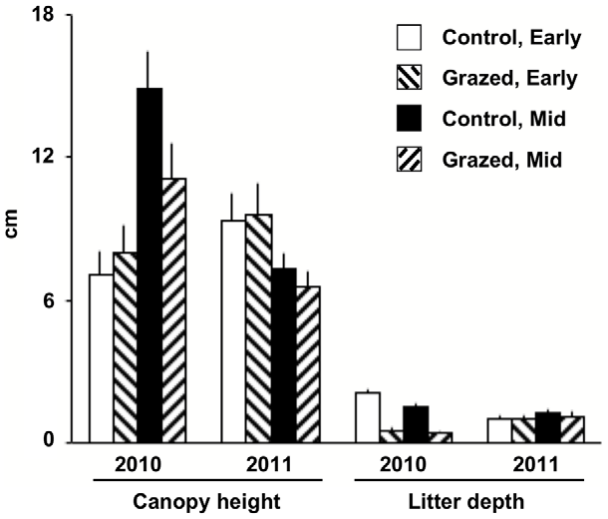
Means (SEs) for canopy height and litter depth by grazing treatment and season. See Table 1 for test results.

A number of vegetation and physical parameters had significant responses to grazing, season, and year, and there were also significant block effects and interaction terms (Table 1, Figs. 1, 2). Litter depth, litter cover, bare ground, and soil strength indicated impacts from the grazing treatment. There was also an overall significant trend of poorer habitat conditions under grazing exposure across metrics, seasons, and years (p = 0.0015; two-tailed sign test; Table 1). Three variables showed seasonal differences: litter cover was lower later in the growing season, whereas brown cover and soil strength (compaction) increased, but most variables had a significant Season x Year interaction term. There was only a single significant Treatment x Season interaction; grazed sites had less brown, senescent cover in early season than control sites, but more brown cover in mid-season (Table 1, Fig. 1). Block effects were present for all variables except bare ground (Table 1).

**Table 1:** Means (standard errors) for vegetation and physical parameters as a function of Treatment (Control, Grazed), Season (Early, Mid), and Year (2010, 2011) and results of 2×2×2 blocked ANCOVAs with elevation as a covariate.

		Early		Mid		ANCOVA							
		Control	Grazed	Control	Grazed	T^a^	S^b^	Y^c^	TxS	TxY	SxY	B^d^	
Canopy height (cm)	'10^e^	7.10 (1.0)	7.98 (1.2)	14.9 (1.6)	11.1 (1.5)						**	*	
	'11^f^	9.33 (1.2)	9.55 (1.4)	7.30 (0.73)	6.54 (0.73)								
Litter depth (cm)	'10	2.08 (0.17)	0.469 (0.23)	1.53 (0.18)	0.393 (0.13)	**				**	*	**	
	'11	1.04 (0.17)	1.00 (0.21)	1.25 (0.18)	1.13 (0.19)								
Litter cover (%)	'10	22.8 (4.0)	10.9 (3.2)	12.5 (3.4)	0.714 (0.46)	**	**			**	*	**	
	'11	10.8 (2.4)	10.4 (3.3)	8.25 (2.0)	6.50 (2.3)								
Bare ground (%)	'10	4.50 (1.1)	22.5 (3.1)	1.25 (0.56)	17.1 (5.4)	*				**	*		
	'11	9.00 (1.9)	9.17 (2.0)	9.25 (1.5)	12.0 (5.2)								
Brown cover (%)	'10	14.5 (2.8)	5.94 (3.2)	10.8 (2.1)	13.2 (3.7)		**	**	**			*	
	'11	12.8 (1.8)	6.67 (1.9)	18.0 (3.0)	27.5 (7.5)								
Green cover (%)	'10	58.3 (6.3)	60.6 (7.5)	75.5 (3.6)	68.9 (6.6)						**	**	
	'11	67.5 (5.0)	73.8 (5.7)	64.5 (4.2)	54.0 (7.9)								
Soil strength (kg/cm^2^)	'10	0.967 (0.10)	1.41 (0.12)	1.52 (0.15)	1.96 (0.13)	**	**	**				**	
	'11	1.71 (0.18)	2.19 (0.20)	2.01 (0.084)	2.73 (0.42)								
Wind speed (km/hr)	'10	7.39 (1.4)	4.66 (1.4)	5.50 (0.69)	3.71 (1.1)							*	
	'11	6.35 (0.95)	4.10 (0.91)	5.90 (1.0)	5.12 (1.1)								
Air temperature (°C)	'10	17.4 (0.77)	18.3 (0.63)	19.6 (1.2)	18.7 (1.8)							*	
	'11	19.8 (0.41)	21.3 (0.85)	17.7 (0.77)	18.8 (0.63)								

a Treatment.

b Season.

c Year.

d Block.

e 2010.

f 2011.

* p<0.05 before sequential Bonferroni correction.

** p<0.05 after sequential Bonferroni correction.

### Fauna

We identified 9,633 arthropods representing 99 families (Table S1) and 239 morphospecies; 63% of the families occurred in three or more samples. Diptera (flies) and Hemiptera (true bugs and leafhoppers) were dominant across all site categories; the most abundant families included ephydrid (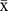 = 39.2/50 sweeps, SE  = 12.5), muscid (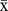 = 22.2, SE  = 3.0), and anthomyiid (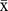 = 13.0, SE  = 1.9) flies and cicadellid (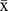 = 28.6, SE  = 4.9) and delphacid (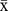 = 11.4, SE  = 3.0) leafhoppers. Family and morphospecies richness were highest for Diptera (37 and 92, respectively), followed by Hymenoptera (wasps, 17, 64), Hemiptera (14, 37), and Coleoptera (beetles, 12, 18; Table S1). Cicadellids and braconid and pteromalid wasps were the families with the largest number of morphospecies (21, 17, and 17, respectively). Relatively motile taxa represented 88% of the assemblage, and there were about twice as many herbivores as predators (Table 2).

**Table 2:** Means (standard errors) for faunal assemblage variables as a function of Treatment (Control, Grazed), Season (Early, Mid), and Year (2010, 2011) and results of 2×2×2 blocked ANCOVAs with elevation as a covariate.

		Early		Mid		ANCOVA							
		Control	Grazed	Control	Grazed	T^a^	S^b^	Y^c^	TxS	TxY	SxY	B^d^	
Total individuals	'10^e^	120 (32)	138 (47)	121 (23)	215 (114)								
	'11^f^	146 (15)	190 (58)	148 (32)	106 (36)								
Family richness	'10	15.4 (2.3)	15.6 (1.9)	15.8 (2.1)	15.1 (2.1)			**					
	'11	23.1 (0.89)	21.8 (2.2)	21.4 (2.1)	19.2 (2.7)								
Morphospecies richness	'10	21.6 (3.3)	21.3 (3.3)	22.1 (3.6)	21.1 (3.7)			**					
	'11	31.1 (1.2)	30.8 (3.5)	33.7 (4.2)	26.8 (5.3)								
Expected number of morphospecies	'10	5.27 (0.29)	5.01 (0.26)	4.49 (0.51)	4.29 (0.82)			**			*		
	'11	5.54 (0.33)	5.97 (0.12)	6.15 (0.26)	6.11 (0.31)								
Probability of interspecific encounter	'10	0.827 (0.026)	0.805 (0.022)	0.676 (0.083)	0.615 (0.12)			**			*		
	'11	0.833 (0.039)	0.892 (0.0073)	0.890 (0.20)	0.885 (0.021)								
% Family dominance	'10	34.0 (3.7)	37.3 (4.0)	48.4 (8.1)	54.9 (11)			**					
	'11	34.0 (5.3)	27.7 (3.1)	30.6 (3.4)	26.3 (4.0)								
% Species dominance	'10	32.3 (3.4)	34.5 (3.5)	47.8 (8.2)	53.7 (11)			**			*		
	'11	31.7 (5.5)	23.5 (2.5)	23.1 (4.2)	24.6 (4.3)								
% Predators	'10	7.26 (1.3)	6.43 (1.2)	10.3 (2.2)	9.23 (3.5)			**					
	'11	9.54 (1.5)	10.9 (2.2)	12.2 (1.5)	15.8 (3.3)								
% Herbivores	'10	29.6 (6.0)	30.0 (8.8)	10.5 (3.2)	8.56 (2.7)			**			**		
	'11	20.2 (4.4)	26.4 (6.4)	33.7 (4.4)	47.2 (7.5)								
% More motile taxa	'10	87.8 (4.4)	91.2 (3.2)	94.3 (1.5)	92.0 (3.4)								
	'11	87.4 (2.6)	88.4 (4.1)	79.9 (3.7)	87.4 (3.8)								
% Less motile taxa	'10	12.1 (4.3)	8.76 (3.2)	5.67 (1.5)	8.00 (3.4)			*					
	'11	12.5 (2.6)	11.6 (4.1)	20.1 (3.8)	12.6 (3.8)								
Baits: Formicidae	'10	0.800 (0.25)	0.875 (0.23)	0.200 (0.13)	0.714 (0.18)		**					**	
species richness	'11	0.600 (0.22)	1.00 (0.37)	0.200 (0.13)	0.600 (0.40)								

All metrics were based on 50-sweep samples, with the exception of ant species richness, which was the result of one aggregate hour of bait deployment using one honey and one tuna bait.

a Treatment.

b Season.

c Year.

d Block.

e 2010.

f 2011.

* p<0.05 before sequential Bonferroni correction.

** p<0.05 after sequential Bonferroni correction.

**Figure 3:**
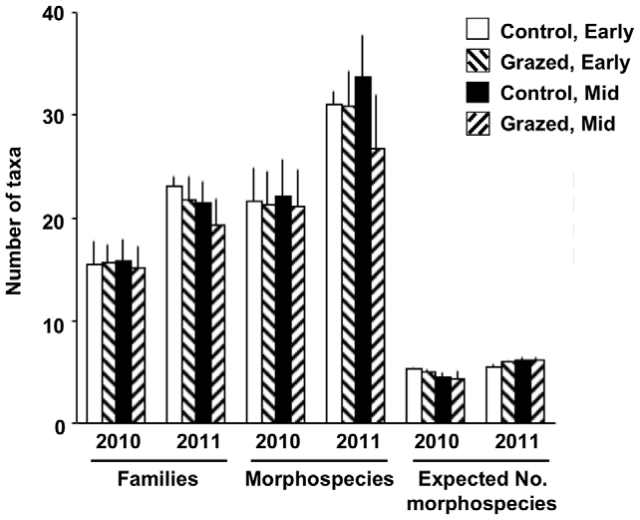
Means (SEs) for families, morphospecies, and expected number of morphospecies by grazing treatment and season. See Table 2 for test results.

**Figure 4:**
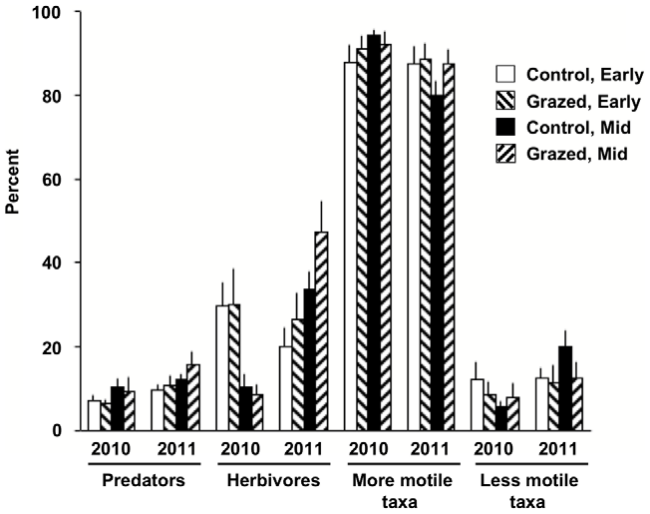
Means (SEs) for predators, herbivores, and more- and less-motile taxa by grazing treatment and season. See Table 2 for test results.

In contrast to vegetation and related variables, fauna showed limited response to both grazing and season at the assemblage level (Table 2, Figs. 3, 4). There were no significant results among assemblage variables for Treatment, and there was not a directional trend across metrics (p = 0.41; two-tailed sign test). Further, there were no significant interactions with either Season or Year. When mid-season plots were examined in isolation, there was a weak trend (p = 0.049) of more depauperate assemblage characteristics on grazed plots among canopy fauna (i.e., bait variable excluded; otherwise p = 0.096). Season effects were only present for ant species richness, but there were some Season x Year interactions, particularly for percentage of herbivores (Table 1, Fig. 4). Year effects, however, occurred across almost all parameters: diversity was higher in 2011 than in 2010 (Table 1, Fig. 3). Year effects were most striking for species dominance at mid-season; 2011 dominance was less than half that of 2010. Block effects, again in contrast to vegetation results, were only present for ant species richness.

There were more seasonal effects at the order and family level, but there were again few grazing effects apparent (Table S2). Three significant Treatment effects were present: for cicadellid leafhoppers, muscid flies, and spiders, and all three taxa had fewer representatives on grazed wetlands than on the paired control wetlands. There was no Treatment trend across all collected families for either early season (p = 0.90; two-tailed sign test; Table S1) or mid-season (p = 0.20). Although there was similarly no Treatment trend across morphospecies in early season (p = 0.76), there was a trend of lower abundance across all morphospecies on grazed plots in mid-season (p = 0.0023). There were again no Treatment x Season interactions and only three significant Treatment x Year interactions without consistent directionality (Table S2). Significant Season effects generally indicated higher abundances in early season. There were numerous Season x Year interactions as well, which were strongest for ephydrid shore flies: abundances were fourteen times greater in mid-season than in early season in 2010, but twice as high in early season as in mid-season in 2011. We collected 85 total families in early season versus 75 in mid-season. Hemiptera generally had highest abundances in early season of 2010 and mid-season of 2011, whereas Diptera showed the inverse relationship (Table S2). Almost half of the abundant taxa showed Year effects, and all of these taxa had higher abundances in 2011, with the exception of bait Formicidae (ants) and the most abundant ant species, *Myrmica discontinua* Weber. Aphids showed the strongest year effects: 28-fold higher abundance in 2011 than in 2010. There were block effects only for leafhoppers and bait Formicidae (Table S2).

There was little clustering by different Treatment and Season combinations for family data (Fig. S1). The initial MRPP randomization test using study factors (T = −2.64, p = 0.0083) did indicate that some treatment combinations were distinct compositionally, but the low within-group agreement value, or effect size (A = 0.045), also suggested substantial variation within each combination. The only significant pairwise comparisons were Early Control vs. Mid Control (p = 0.020) and Early Control vs. Mid Grazed (p = 0.0016). The second MRPP using the new group membership variable generated from cluster analysis resulted in a lower p value (T = −9.79, p<0.0001) and higher A ( = 0.24), and all pairwise comparisons were significant (p<0.024) emphasizing the potential importance of other influences in addition to Treatment and Season. The overall dispersion analysis did not yield significant results. Results were consistent, whether interpreted on the basis of deviations from centroids or from spatial medians (in each case from both ANOVA tables and permutation of least squares residuals); p-values ranged from 0.86 to 0.90. None of the pairwise tests for differences in dispersion among groups were significant (0.42<p<0.92). The non-significant dispersion results in combination with the significant MRPP results, albeit with low effect size, suggest that assemblages did differ compositionally at the family level rather than in variability/dispersion.

There was similarly little clustering at the morphospecies level (Fig. S2). As with family data, the initial MRPP based on morphospecies was significant (T = −3.72, p = 0.00043), but effect size was again low (A = 0.069). Three pairwise comparisons were significant: Early vs Mid Control (p = 0.00019), Early Control vs. Mid Grazed (p = 0.0060), and Early Grazed vs. Mid Control (p = 0.0010). The strongest MRPP results in the study were obtained for morphospecies data in combination with the new group membership variable written from the cluster analyses (T = −11.9, p<0.0001), although effect size was the same as for families (A = 0.24). All of the ensuing pairwise comparisons were significant (p<0.0017). There were no significant results from the overall dispersion analyses (0.12<p<0.23), regardless of derivation from centroids or spatial medians (or ANOVA tables or permutation of residuals), and there were no significant pairwise results (0.13<p<0.86).

## Discussion

There were moderate long-term grazing effects on subalpine wetland vegetation, but few negative indirect effects on fauna, and these relationships changed only subtly at mid-season. Although long-term vs. short-term differences in grazing effects have been documented elsewhere [1], Treatment x Season interactions were in fact the least common of all the examined sources of variance in our study (only one significant interaction). The only negative effects on fauna were: three Treatment differences for individual taxa (of 32 tests) that indicated lower numbers on grazed plots, weakly-significant lower diversity on mid-season grazed sites across canopy assemblage metrics, and a significant trend of lower abundances on mid-season grazed sites across all morphospecies. These effects, though certainly minimal, do differ somewhat from results of the early season-only study that detected no effects of grazing on fauna, the exceptions being a small number of positive effects [12]. Nevertheless, it seems clear that the lack of long-term effects was not obscuring major impacts on either flora or fauna manifesting at mid-season, thus corroborating the overall conclusion of the earlier study: little negative impact of grazing on fauna.

The subalpine vegetation metrics negatively influenced by grazing disturbance in our study have often been shown to be affected in other, more intensively grazed, environments [53], [67]–[69]. Effects on bare ground and litter are sufficiently established that these characteristics have been used as proxies for grazing intensity and/or as manipulated variables used in experimental assessments of grazing pressure [52], [53], [70]. Litter removal can cause shifts in soil moisture [70] which could, along with direct pressure from trampling, mediate some of the observed increases in soil strength (in the form of compaction) on our sites. The significant interaction for senescent, brown cover (less common on grazed than on control sites in early season but more common on grazed sites in mid-season) may have been driven by a) breakage of the less flexible standing senescent vegetation that dominates these wet meadows in late season [33] with effects that remained through the ensuing winter and into the next growing season (see also [1], [12]), and b) mid-season impacts, such as urine patches, that may temporarily increase standing dead vegetation [71], [72] in otherwise non-senescent wetlands.

Wetland arthropods are often sensitive to disturbance [73], and livestock grazing can negatively affect arthropods [24], [26], [74], so sufficiently strong grazing pressure would have been expected to produce effects on this arthropod assemblage. Grazing can affect arthropods indirectly via effects on litter and structural diversity [69], [75], [76], such as we observed in our grazed wetlands. Because of such effects on vegetation, herbivores [75], [77], particularly leafhoppers, can be sensitive to livestock grazing ([27]; but see [74]), and indeed these abundant animals were the group most affected by grazing in our study. Grazing can alternatively have neutral or positive indirect effects on arthropods [50], [67], [78] despite negative effects on vegetation [12], [68]. The minor negative effects of grazing on fauna in the current study indicate that pack stock disturbance in Sequoia does not fall to either extreme of this spectrum of effects, and the subtle indications of additional mid-season grazing effects most closely align with studies that have found only limited divergence between long- and short-term grazing effects on ecosystems [2]–[4].

A variety of factors may have contributed to both the lack of overall effects of grazing disturbance on fauna and the minor additional grazing effect apparent during the growing season. Stock usage of these wetlands is relatively low and is prohibited during the most vulnerable period immediately following snowmelt ([12]; see also [33]). Stock usage was further reduced during the two study years as a result of late wetland openings due to heavy preceding winters. The early-season wetland closures in the Park made the study possible by allowing examination of early-season effects in isolation, but earlier and more intensive stock use in other managed areas would likely produce greater effects. Sierran wet meadows may also be resistant to grazing as a result of decades-long use (see also [16], [33]), possibly reducing, but clearly not eliminating, effects on vegetation structure. Resistance to pack stock may further vary as a function of vegetation assemblage [18], [33]; the reed grass-dominated assemblage, frequently used by packers and emphasized in our study, may tolerate stock better than wetter vegetation assemblages. Faunal differences as a function of grazing may be mitigated by frequent movement of arthropods among habitats ([21]–[23], c.f. [79]) facilitated by permeable micro-landscape boundaries [80]–[82] between grazed and ungrazed habitat, particularly for the motile taxa that dominated the assemblage. A number of taxa have life history stages in other habitats, for instance the subalpine streams typically bisecting these wetlands [83], that may function as refugia from terrestrial disturbance.

Sampling grain can also mediate detection of livestock grazing effects [34], [84]–[86]. Our study was cast at the landscape scale, i.e., the scale of management interest, and the experimental units in our study were entire wetlands [3]. Cropping in our study wetlands was patchy, as might be expected with low stock densities [85], and cropping intensity varied across our randomly selected subsampling areas. Although we believe that our work captured grazing effects at the wetland and landscape scale, greater effects may have been present in small, intensively-grazed patches (see also [34], [85]).

Season and Year had approximately equal influence on overall vegetation structure in these high elevation wetlands, but inter-annual variability was greater than seasonal variability for fauna, particularly at the assemblage level. There was greater faunal diversity and abundance in the particularly wet 2011 than in 2010, and this result is consistent with correlations between these faunal metrics and snow water equivalent over several years in both the Sierra Nevada and White Mountains of California (Holmquist and Schmidt-Gengenbach, unpublished meeting abstract). Clearly, idiosyncrasies of the year or years selected for study can influence conclusions regarding assemblage structure and response to grazing disturbance (see also [3]), as has been demonstrated in other ecosystems (e.g., [6]). Although the early/mid-season differences were of secondary importance for fauna, diversity and abundance decline by about a factor of four with wetland senescence during late season (Holmquist and Schmidt-Gengenbach, unpublished report). The trend of higher abundances for individual taxa in early season may be a function of early season hatching/emergence, or may suggest that soil moisture or the initial arrival of warmer weather is more important than the more fully developed vegetation structure found in mid-season, but our study was not designed to establish mechanisms for seasonal differences. The numerous Season x Year interactions were also likely related to the divergent snow years, and canopy height, green cover, and a number of faunal assemblage and family metrics showed congruent responses.

It appears that current management of pack stock in the Park has produced moderate negative effects on coarse vegetation structure, but only minimal effects on the arthropod assemblage. This study, however, did capture some minor grazing effects at mid-season that were not apparent from early season sampling that targeted persisting effects only [12]. These results, coupled with the moderate disturbance to vegetation, raise the concern that increases in stock usage of these wetlands could cause more significant impact. Conversely, current Park maintenance of low stock densities and attention to wetland soil moisture and plant phenology in determining opening dates likely combine to prevent greater impact, and other land management agencies might benefit from application of these standards. The results of the current study should assist with the resolution of hiker-equestrian conflict associated with pack stock use [32], [35], [87] by demonstrating the complexity of ecosystem response to this disturbance: current stock use appears to be neither completely benign nor vastly destructive. Reliance on either vegetation or faunal metrics exclusively would have yielded different overall conclusions, whereas examining effects though the lenses of both flora and fauna provided a more integrative and nuanced view of ecosystem response.

## Supporting Information

Figure S1 **Agglomerative cluster analysis of site family data with overlay by grazing treatment and season.**
(PPT)

Figure S2 **Agglomerative cluster analysis of site morphospecies data with overlay by grazing treatment and season.**
(PPT)

Table S1 **Mean relative abundance/50 sweeps** (**standard error**)**, both years combined, as a function of grazing and season; zeros are omitted for clarity.**
(DOC)

Table S2 **Abundance means** (**standard errors**) **for orders and ten most abundant families as a function of Treatment** (**Control, Grazed**)**, Season** (**Early, Mid**)**, and Year** (**2010, 2011**) **and results of 2×2×2 blocked ANCOVAs with elevation as a covariate.**
(DOC)
