# Supplementary figures and images for: Effects of a Long-Term Disturbance on Arthropods and Vegetation in Subalpine Wetlands: Manifestations of Pack Stock Grazing in Early versus Mid-Season
Source: PLoS One. 2013 Jan 7;8(1):e54109. doi: 10.1371/journal.pone.0054109 (PMC3538743; doi:10.1371/journal.pone.0054109)

## Slide 1
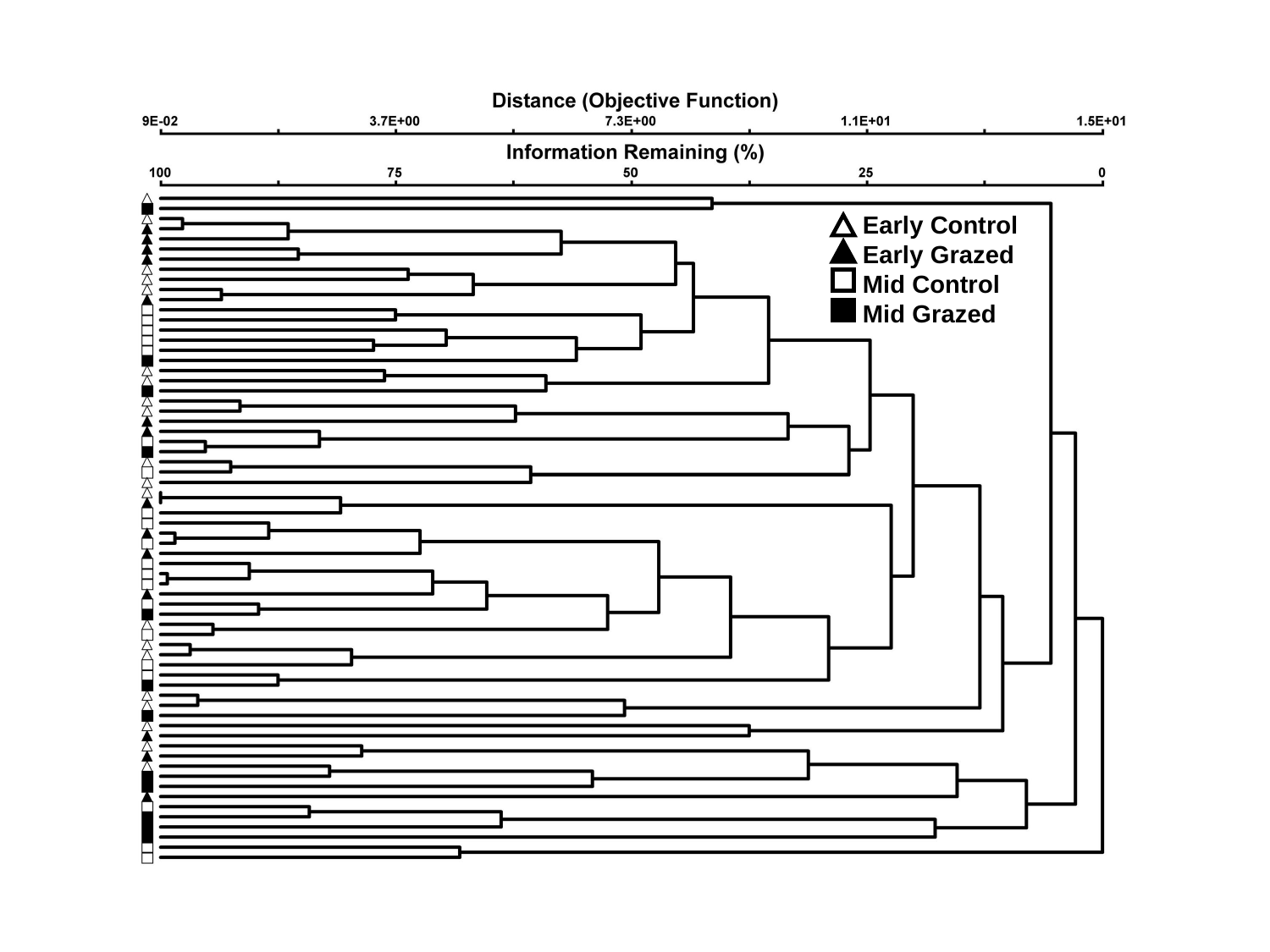

Early Control
Early Grazed
Mid Control
Mid Grazed

Supplement: Figure S1 — Agglomerative cluster analysis of site family data with overlay by grazing treatment and season. (PPT) [file pone.0054109.s001.ppt]

## Slide 1
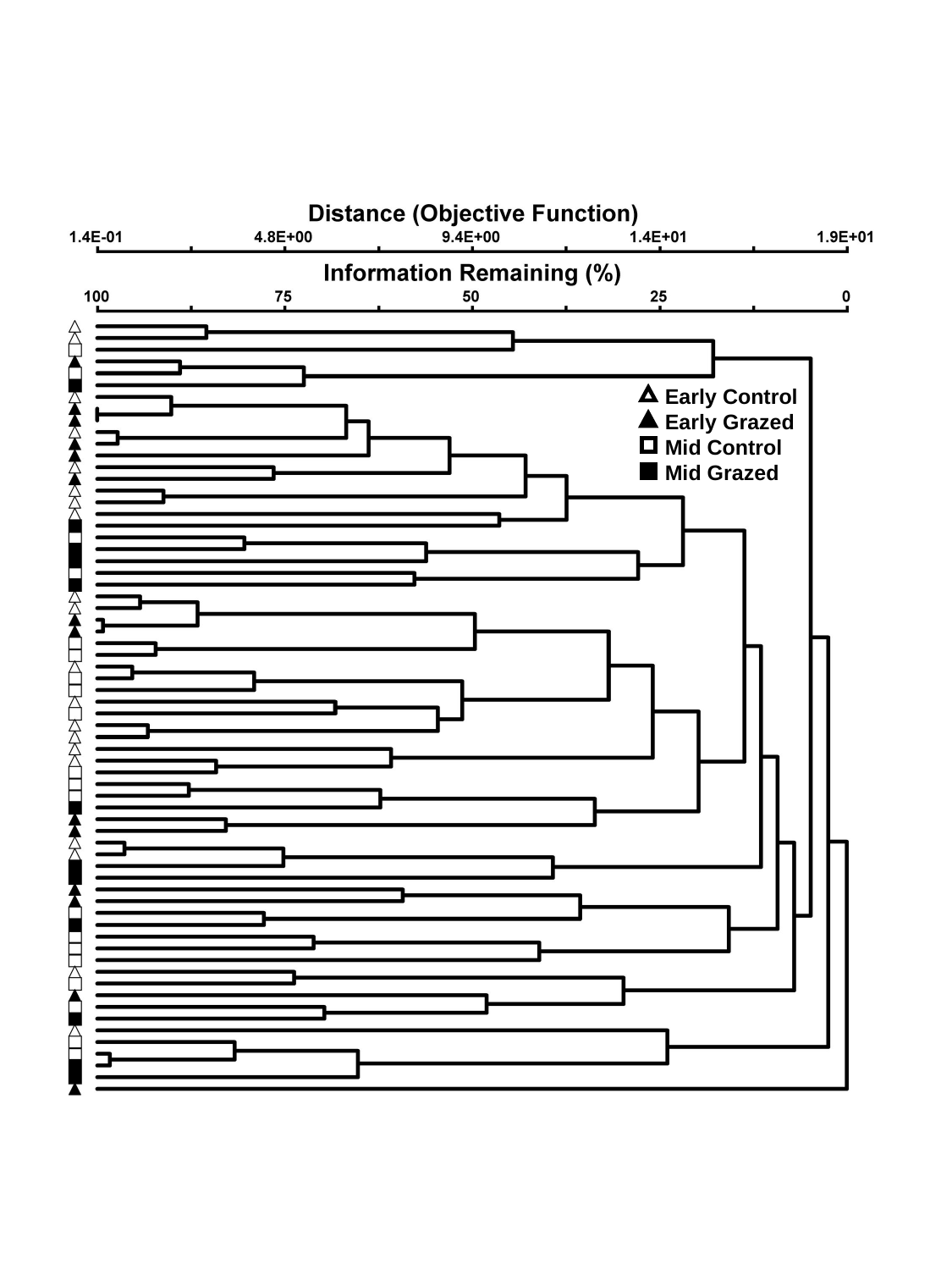

Early Control
Early Grazed
Mid Control
Mid Grazed

Supplement: Figure S2 — Agglomerative cluster analysis of site morphospecies data with overlay by grazing treatment and season. (PPT) [file pone.0054109.s002.ppt]
