# Supplementary material for: Effects of a Long-Term Disturbance on Arthropods and Vegetation in Subalpine Wetlands: Manifestations of Pack Stock Grazing in Early versus Mid-Season
Source: PLoS One. 2013 Jan 7;8(1):e54109. doi: 10.1371/journal.pone.0054109 (PMC3538743; doi:10.1371/journal.pone.0054109)
Supplement: Table S1 — Mean relative abundance/50 sweeps (standard error), both years combined, as a function of grazing and season; zeros are omitted for clarity. (DOC) [file pone.0054109.s003.doc]

**Supporting Information**

**Table S1. Mean relative abundance/50 sweeps (standard error), both years combined, as a function of grazing and season; zeros are omitted for clarity.**

(DOC)

|  | Early | | Mid | |
| --- | --- | --- | --- | --- |
|  | Control | Grazed | Control | Grazed |
| Orthoptera |  |  |  |  |
| Acrididae | 0.350 (0.21) | 1.14 (0.81) | 0.100 (0.069) | 0.750 (0.33) |
| Plecoptera |  |  |  |  |
| Chloroperlidae | 0.0500 (0.050) |  | 0.0500 (0.050) |  |
| Hemiptera |  |  |  |  |
| Saldidae | 0.250 (0.20) |  |  |  |
| Miridae | 0.100 (0.069) | 0.143 (0.097) | 0.800 (0.40) | 0.417 (0.19) |
| Nabidae | 0.100 (0.10) | 0.214 (0.11) | 0.250 (0.12) | 0.167 (0.11) |
| Anthocoridae |  |  | 0.0500 (0.050) |  |
| Scutelleridae |  |  |  | 0.0830 (0.083) |
| Berytidae |  | 0.0710 (0.071) |  |  |
| Rhyparochromidae | 0.100 (0.069) |  |  | 0.0830 (0.083) |
| Lygaeidae | 0.900 (0.29) | 1.00 (0.43) | 0.750 (0.24) | 0.250 (0.13) |
| Geocoridae | 0.0500 (0.050) | 0.143 (0.097) | 0.0500 (0.050) | 0.0830 (0.083) |
| Rhopalidae | 0.150 (0.15) |  | 0.250 (0.18) |  |
| Cicadellidae | 21.0 (5.5) | 32.4 (10) | 17.4 (4.8) | 11.3 (4.2) |
| Delphacidae | 11.7 (7.14) | 25.1 (12) | 3.95 (1.6) | 13.4 (7.3) |
| Psyllidae | 0.100 (0.10) | 0.143 (0.097) | 0.300 (0.13) | 0.167 (0.11) |
| Aphididae | 1.80 (0.99) | 1.43 (0.72) | 7.60 (3.2) | 1.50 (0.73) |
| Thysanoptera |  |  |  |  |
| Thripidae | 0.450 (0.20) | 0.286 (0.22) |  |  |
| Coleoptera |  |  |  |  |
| Dytiscidae |  |  | 0.0500 (0.050) |  |
| Hydraenidae |  | 0.0710 (0.071) |  |  |
| Staphylinidae | 0.250 (0.20) | 0.214 (0.11) | 0.200 (0.12) | 0.167 (0.11) |
| Cantharidae | 0.100 (0.069) | 0.214 (0.11) |  |  |
| Dermestidae |  | 0.143 (0.14) |  |  |
| Cleridae |  | 0.0710 (0.071) |  |  |
| Melyridae | 0.150 (0.082) | 0.0710 (0.071) | 0.0500 (0.050) |  |
| Coccinellidae | 0.0500 (0.050) |  | 0.100 (0.069) | 0.250 (0.25) |
| Mordellidae | 0.250 (0.12) | 0.0710 (0.071) | 0.400 (0.18) | 0.167 (0.11) |
| Anthicidae | 0.0500 (0.050) |  |  |  |
| Chrysomelidae |  |  |  | 0.0830 (0.083) |
| Curculionidae | 0.100 (0.069) |  |  |  |
| Hymenoptera |  |  |  |  |
| Xyelidae | 0.0500 (0.050) |  |  |  |
| Tenthredinidae |  | 0.0710 (0.071) |  |  |
| Braconidae | 0.950 (0.27) | 0.714 (0.24) | 1.35 (0.35) | 0.417 (0.19) |
| Ichneumonidae | 1.10 (0.40) | 0.786 (0.26) | 1.05 (0.29) | 0.333 (0.19) |
| Pteromalidae | 0.400 (0.13) | 1.57 (0.83) | 2.10 (0.86) | 1.83 (1.3) |
| Eurytomidae | 0.0500 (0.050) |  |  |  |
| Chalcididae |  |  | 0.0500 (0.050) |  |
| Eucoilidae | 0.0500 (0.050) |  | 0.300 (0.13) |  |
| Cynipidae |  |  | 0.0500 (0.050) |  |
| Diapriidae | 0.150 (0.11) | 0.0710 (0.071) | 0.0500 (0.050) | 0.250 (0.25) |
| Chrysididae | 0.0500 (0.050) |  |  |  |
| Bethylidae |  |  |  | 0.0830 (0.083) |
| Dryinidae |  |  | 0.0500 (0.050) |  |
| Halictidae | 0.0500 (0.050) | 0.0710 (0.071) |  | 0.167 (0.17) |
| Megachilidae |  | 0.0710 (0.071) |  |  |
| Apidae |  |  | 0.0500 (0.050) |  |
| Formicidae | 1.00 (0.60) | 5.57 (4.7) | 0.600 (0.31) | 1.00 (0.56) |
| Lepidoptera |  |  |  |  |
| Coleophoridae |  | 0.143 (0.097) | 0.100 (0.069) | 0.0830 (0.083) |
| Tortricidae | 0.0500 (0.050) |  |  |  |
| Pyralidae |  | 0.0710 (0.071) |  | 0.0830 (0.083) |
| Crambidae |  |  |  |  |
| Noctuidae | 1.30 (0.54 ) | 0.429 (0.29) | 0.150 (0.082) |  |
| Diptera |  |  |  |  |
| Tipulidae | 0.0500 (0.050) | 0.0710 (0.071) | 0.200 (0.12) |  |
| Ceratopogonidae | 0.750 (0.50) |  | 0.0500 (0.050) | 0.250 (0.25) |
| Chironomidae | 0.400 (0.22) | 0.0710 (0.071) | 1.30 (0.95) | 0.333 (0.33) |
| Culicidae | 2.40 (1.4) | 2.29 (1.1) | 0.0500 (0.050) | 0.0830 (0.083) |
| Simuliidae | 0.0500 (0.050) |  | 0.200 (0.16) |  |
| Bibionidae | 0.100 (0.069) |  |  | 0.0830 (0.083) |
| Cecidomyiidae |  |  | 0.150 (0.15) | 0.250 (0.25) |
| Mycetophilidae | 0.200 (0.16) | 0.0710 (0.071) | 0.150 (0.15) | 0.167 (0.17) |
| Sciaridae | 4.75 (3.2) | 1.43 (0.58) | 1.95 (0.59) | 0.750 (0.35) |
| Athericidae |  | 0.0710 (0.071) |  |  |
| Rhagionidae |  | 0.0710 (0.071) |  |  |
| Asilidae | 0.0500 (0.050) |  |  |  |
| Therevidae | 0.0500 (0.050) |  |  |  |
| Empididae | 2.00 (0.86) | 3.57 (2.1) | 1.15 (0.37) | 0.833 (0.53) |
| Dolichopodidae | 1.05 (0.43) | 2.29 (0.95) | 1.95 (0.96) | 0.500 (0.42) |
| Lonchopteridae | 0.500 (0.24) | 0.643 (0.36) |  | 0.0830 (0.083) |
| Phoridae | 0.400 (0.13) | 1.14 (0.53) | 0.500 (0.35) | 1.25 (0.76) |
| Pipunculidae | 0.250 (0.14) | 0.143 (0.097) | 0.250 (0.18) | 0.167 (0.11) |
| Syrphidae | 0.200 (0.16) | 0.143 (0.097) | 0.0500 (0.050) | 0.333 (0.14) |
| Anthomyiidae | 10.5 (2.1) | 10.0 (1.7) | 14.6 (3.9) | 19.5 (9.9) |
| Muscidae | 33.6 (6.1) | 37.4 (9.4) | 18.3 (6.4) | 4.67 (1.1) |
| Sarcophagidae | 0.100 (0.069) |  | 0.0500 (0.050) |  |
| Scathophagidae | 0.55 0 (0.25) |  | 1.20 (0.41) | 0.167 (0.11) |
| Tachinidae | 0.300 (0.13) | 0.0710 (0.071) | 0.350 (0.17) | 0.167 (0.11) |
| Psilidae | 0.150 (0.11) |  | 0.0500 (0.050) |  |
| Lonchaeidae |  |  | 0.300 (0.16) | 0.333 (0.26) |
| Tephritidae | 0.300 (0.13) | 0.429 (0.17) | 2.35 (1.5) | 0.417 (0.23) |
| Chamaemyiidae |  | 0.643 (0.57) |  | 0.250 (0.25) |
| Sepsidae | 2.95 (1.5) | 2.14 (1.3) | 1.15 (0.61) | 1.17 (0.76) |
| Agromyzidae | 1.85 (0.33) | 1.86 (0.83) | 3.95 (1.2) | 2.42 (0.61) |
| Opomyzidae | 0.0500 (0.050) |  |  | 0.167 (0.11) |
| Chloropidae | 6.10 (2.0) | 6.57 (1.5) | 3.75 (0.94) | 2.33 (0.47) |
| Tethinidae | 0.0500 (0.050) |  |  |  |
| Heleomyzidae | 0.900 (0.30) | 0.286 (0.16) | 0.700 (0.39) | 1.83 (1.3) |
| Sphaeroceridae | 1.75 (0.68) | 2.64 (1.6) | 2.15 (0.73) | 1.50 (0.65) |
| Drosophilidae | 1.15 (0.55) | 1.14 (0.59) | 1.00 (0.41) | 2.17 (0.82) |
| Ephydridae | 12.0 (3.6) | 7.93 (2.6) | 32.3 (13) | 90.0 (61) |
| Araneae |  |  |  |  |
| Araneidae | 1.40 (0.42) | 1.93 (1.1) | 2.35 (0.41) | 2.67 (1.4) |
| Tetragnathidae | 0.550 (0.20) | 0.0710 (0.071) | 0.0500 (0.050) |  |
| Linyphiidae | 0.850 (0.37) | 0.857 (0.29) | 1.050 (0.80) | 0.333 (0.14) |
| Dyctinidae | 0.0500 (0.050) | 0.0710 (0.071) | 0.0500 (0.050) |  |
| Lycosidae | 0.0500 (0.050) |  | 0.100 (0.10) |  |
| Thomisidae | 1.35 (0.43) | 1.57 (0.69) | 2.65 (1.0) | 0.917 (0.26) |
| Salticidae |  |  | 0.0500 (0.050) |  |
| Acari |  |  |  |  |
| Thrombidiidae |  | 0.0710 (0.071) |  |  |
