# Supplementary material for: Effects of a Long-Term Disturbance on Arthropods and Vegetation in Subalpine Wetlands: Manifestations of Pack Stock Grazing in Early versus Mid-Season
Source: PLoS One. 2013 Jan 7;8(1):e54109. doi: 10.1371/journal.pone.0054109 (PMC3538743; doi:10.1371/journal.pone.0054109)
Supplement: Table S2 — Abundance means (standard errors) for orders and ten most abundant families as a function of Treatment (Control, Grazed), Season (Early, Mid), and Year (2010, 2011) and results of 2×2×2 blocked ANCOVAs with elevation as a covariate. (DOC) [file pone.0054109.s004.doc]

**Table S2. Abundance means (standard errors) for orders and ten most abundant families as a function of Treatment (Control, Grazed), Season (Early, Mid), and Year (2010, 2011) and results of 2x2x2 blocked ANCOVAs with elevation as a covariate.**

(DOC)

|  |  | Early | | Mid | | ANCOVA | | | | | | |
| --- | --- | --- | --- | --- | --- | --- | --- | --- | --- | --- | --- | --- |
|  |  | Control | Grazed | Control | Grazed | T^a^ | S^b^ | Y^c^ | TxS | TxY | SxY | B^d^ |
| Orthoptera | '10^e^ | 0.200 (0.13) | 0.125 (0.13) | 0.100 (0.10) | 0.286 (0.18) |  |  | ** |  | * |  |  |
|  | '11^f^ | 0.500 (0.40) | 2.50 (1.8) | 0.100 (0.10) | 1.40 (0.68) |  |  |  |  |  |  |  |
| Plecoptera | '10 | 0.100 (0.10) | 0.0 (0.0) | 0.0 (0.0) | 0.0 (0.0) | ID^g^ |  |  |  |  |  |  |
|  | '11 | 0.0 (0.0) | 0.0 (0.0) | 0.100 (0.10) | 0.0 (0.0) |  |  |  |  |  |  |  |
| Hemiptera | '10 | 42.4 (23) | 59.1 (29) | 9.00 (2.9) | 6.86 (2.5) |  | * | ** |  |  | ** | * |
|  | '11 | 30.1 (8.7) | 62.7 (28) | 53.8 (14) | 58.0 (25) |  |  |  |  |  |  |  |
| Cicadellidae | '10 | 23.7 (9.3) | 23.1 (9.3) | 5.90 (1.7) | 4.43 (1.7) | ** | ** | ** |  |  | ** | ** |
|  | '11 | 18.2 (6.4) | 44.7 (20) | 28.9 (8.1) | 21.0 (8.4) |  |  |  |  |  |  |  |
| Delphacidae | '10 | 17.5 (14) | 33.8 (20) | 1.10 (0.89) | 1.57 (1.0) |  |  | * |  |  | ** | * |
|  | '11 | 5.80 (2.8) | 13.5 (6.7) | 6.80 (2.8) | 30.0 (15) |  |  |  |  |  |  |  |
| Aphididae | '10 | 0.100 (0.10) | 0.250 (0.16) | 0.400 (0.22) | 0.143 (0.14) |  | * | ** |  |  | * |  |
|  | '11 | 3.50 (1.9) | 3.00 (1.5) | 14.8 (5.7) | 3.40 (1.4) |  |  |  |  |  |  |  |
| Thysanoptera | '10 | 0.300 (0.30) | 0.0 (0.0) | 0.0 (0.0) | 0.0 (0.0) |  | ** | * |  |  | * |  |
|  | '11 | 0.900 (0.35) | 0.667 (0.49) | 0.0 (0.0) | 0.0 (0.0) |  |  |  |  |  |  |  |
| Coleoptera | '10 | 0.500 (0.22) | 0.625 (0.26) | 0.200 (0.13) | 0.571 (0.43) |  |  | ** |  |  |  |  |
|  | '11 | 1.40 (0.48) | 1.17 (0.48) | 1.40 (0.40) | 0.800 (0.37) |  |  |  |  |  |  |  |
| Hymenoptera | '10 | 3.20 (1.2) | 2.50 (0.73) | 4.90 (1.3) | 2.57 (1.2) |  |  | * |  |  |  |  |
|  | '11 | 4.50 (1.2) | 17.5 (11) | 6.40 (2.3) | 6.20 (2.9) |  |  |  |  |  |  |  |
| Lepidoptera | '10 | 0.900 (0.60) | 0.250 (0.16) | 0.300 (0.15) | 0.286 (0.29) |  | * |  |  |  |  |  |
|  | '11 | 1.80 (0.89) | 1.33 (0.80) | 0.200 (0.13) | 0.0 (0.0) |  |  |  |  |  |  |  |
| Diptera | '10 | 69.1 (16) | 72.5 (19) | 103 (23) | 201 (116) |  |  |  |  |  | ** |  |
|  | '11 | 102 (11) | 97.2 (24) | 77.3 (17) | 34.8 (11) |  |  |  |  |  |  |  |
| Sciaridae | '10 | 7.90 (6.4) | 1.13 (0.72) | 1.70 (0.96) | 0.714 (0.47) |  |  |  |  |  |  |  |
|  | '11 | 1.60 (0.37) | 1.83 (1.0) | 2.20 (0.74) | 0.800 (0.58) |  |  |  |  |  |  |  |
| Anthomyiidae | '10 | 6.30 (1.6) | 9.63 (1.9) | 20.7 (7.2) | 31.9 (16) |  |  |  |  | * | ** |  |
|  | '11 | 14.7 (3.4) | 10.5 (3.4) | 8.40 (2.0) | 2.20 (1.0) |  |  |  |  |  |  |  |
| Muscidae | '10 | 26.1 (5.5) | 37.9 (13) | 8.50 (4.3) | 4.57 (1.7) | * | ** |  |  |  |  |  |
|  | '11 | 41.0 (11) | 36.8 (14) | 28.0 (12) | 4.80 (1.6) |  |  |  |  |  |  |  |
| Agromyzidae | '10 | 1.60 (0.40) | 2.25 (1.4) | 2.20 (0.76) | 1.71 (0.57) |  |  |  |  |  |  |  |
|  | '11 | 2.10 (0.53) | 1.33 (0.84) | 5.70 (2.1) | 3.40 (1.2) |  |  |  |  |  |  |  |
| Chloropidae | '10 | 6.90 (3.7) | 6.38 (2.2) | 3.70 (1.4) | 2.14 (0.60) |  |  |  |  |  |  |  |
|  | '11 | 5.30 (1.6) | 6.83 (2.1) | 3.80 (1.3) | 2.60 (0.81) |  |  |  |  |  |  |  |
| Ephydridae | '10 | 3.90 (1.3) | 3.13 (1.3) | 54.4 (25) | 149 (101) |  | * |  |  |  | ** |  |
|  | '11 | 20.1 (6.2) | 14.3 (5.0) | 10.0 (3.6) | 6.80 (4.5) |  |  |  |  |  |  |  |
| Araneae | '10 | 3.10 (1.1) | 3.13 (1.1) | 3.70 (1.8) | 3.14 (0.67) | * |  |  |  |  |  |  |
|  | '11 | 5.30 (0.98) | 6.33 (3.5) | 9.00 (2.3) | 5.00 (3.8) |  |  |  |  |  |  |  |
| Araneidae | '10 | 0.200 (0.13) | 1.13 (0.61) | 1.60 (0.54) | 1.86 (0.46) |  |  | * |  | ** |  |  |
|  | '11 | 2.60 (0.64) | 3.00 (2.4) | 3.10 (0.55) | 3.80 (3.6) |  |  |  |  |  |  |  |
| Acari | '10 | 0.0 (0.0) | 0.125 (0.13) | 0.0 (0.0) | 0.143 (0.14) |  |  |  |  |  |  |  |
|  | '11 | 0.0 (0.0) | 0.0 (0.0) | 0.0 (0.0) | 0.0 (0.0) |  |  |  |  |  |  |  |
| Baits |  |  |  |  |  |  |  |  |  |  |  |  |
| *Myrmica discontinua* | '10 | 4.40 (2.7) | 3.00 (1.9) | 0.600 (0.40) | 3.57 (1.8) |  |  | * |  |  |  |  |
|  | '11 | 1.00 (0.60) | 1.50 (0.76) | 0.100 (0.10) | 0.800 (0.58) |  |  |  |  |  |  |  |
| Total Formicidae | '10 | 4.90 (2.7) | 3.38 (1.8) | 0.600 (0.40) | 5.14 (1.9) |  |  | * |  |  |  | * |
|  | '11 | 1.30 (0.58) | 2.00 (0.78) | 0.200 (0.13) | 2.20 (2.0) |  |  |  |  |  |  |  |
| Total Acari | '10 | 0.600 (0.40) | 1.00 (0.42) | 0.100 (0.10) | 0.0 (0.0) |  |  |  |  |  | * |  |
|  | '11 | 0.0 (0.0) | 0.167 (0.17) | 0.0 (0.0) | 0.600 (0.60) |  |  |  |  |  |  |  |

All metrics were based on 50-sweep samples, with the exception of bait variables, which were the result of one aggregate hour of bait deployment using one honey and one tuna bait.

^a^Treatment

^b^Season

^c^Year

^d^Block

^e^2010

^f^2011

^g^Insufficient Plecoptera data for testing

* p < 0.05 before sequential Bonferroni correction

** p < 0.05 after sequential Bonferroni correction
